# Referenceless 4D flow cardiovascular magnetic resonance with deep learning

**DOI:** 10.1016/j.jocmr.2025.101920

**Published:** 2025-06-02

**Authors:** Chiara Trenti, Erik Ylipää, Tino Ebbers, Carl-Johan Carlhäll, Jan Engvall, Petter Dyverfeldt

**Affiliations:** a Department of Health, Medicine and Caring Sciences (HMV), Linköping University, Linköping, Sweden; b Center for Medical Image Science and Visualization (CMIV), Linköping, Sweden; c Analytic Imaging Diagnostics Arena (AIDA), Linköping University, Linköping, Sweden; d Science for Life Laboratory, Linköping University, Linköping, Sweden; e Department of Clinical Physiology in Linköping, and Department of Health, Medicine and Caring Sciences (HMV), Linköping University, Linköping, Sweden

**Keywords:** 4D flow, Phase-contrast, Deep learning, Accelerated techniques, Cardiovascular imaging

## Abstract

**Background:**

Despite its potential to improve the assessment of cardiovascular diseases, four-dimensional (4D) flow cardiovascular magnetic resonance (CMR) is hampered by long scan times. 4D flow CMR is conventionally acquired with three motion encodings and one reference encoding, as the three-dimensional velocity data are obtained by subtracting the phase of the reference from the phase of the motion encodings. In this study, we aim to use deep learning to predict the reference encoding from the three motion encodings for cardiovascular 4D flow.

**Methods:**

A U-Net was trained with adversarial learning (U-Net_ADV_) and with a velocity frequency-weighted loss function (U-Net_VEL_) to predict the reference encoding from the three motion encodings obtained with a non-symmetric velocity-encoding scheme. Whole-heart 4D flow datasets from 126 patients with different types of cardiomyopathies were retrospectively included. The models were trained on 113 patients with a 5-fold cross-validation, and tested on 13 patients. Flow volumes in the aorta and pulmonary artery, mean and maximum velocity, total and maximum turbulent kinetic energy at peak systole in the cardiac chambers and main vessels were assessed.

**Results:**

Three-dimensional velocity data reconstructed with the reference encoding predicted by deep learning agreed well with the velocities obtained with the reference encoding acquired at the scanner for both models. U-Net_ADV_ performed more consistently throughout the cardiac cycle and across the test subjects, while U-Net_VEL_ performed better for systolic velocities. Comprehensively, the largest error for flow volumes, maximum and mean velocities was −6.031% for maximum velocities in the right ventricle for the U-Net_ADV_, and −6.92% for mean velocities in the right ventricle for U-Net_VEL_. For total turbulent kinetic energy, the highest errors were in the left ventricle (−77.17%) for the U-Net_ADV_, and in the right ventricle (24.96%) for the U-Net_VEL_, while for maximum turbulent kinetic energy were in the pulmonary artery for both models, with a value of −15.5% for U-Net_ADV_ and 15.38% for the U-Net_VEL_.

**Conclusion:**

Deep learning-enabled referenceless 4D flow CMR permits velocities and flow volumes quantification comparable to conventional 4D flow. Omitting the reference encoding reduces the amount of acquired data by 25%, thus allowing shorter scan times or improved resolution, which is valuable for utilization in the clinical routine.

## Graphical abstract

**Figure:**
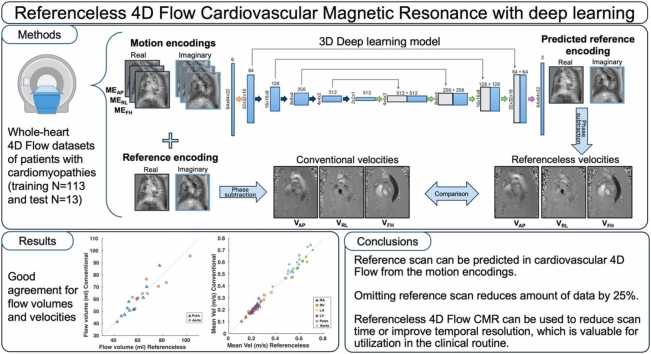

## 1. Introduction

Cine three-dimensional phase-contrast cardiovascular magnetic resonance (four-dimensional [4D] flow CMR) permits non-invasive comprehensive quantification of blood flow in the cardiac chambers and vasculature [1]. Cardiovascular blood flow assessment with 4D flow CMR is clinically useful in various anatomies and diseases, such as aortic aneurysms, dissection, and congenital heart disease [2], [3], [4], [5]. The potential of 4D flow CMR has become evident, not only in improving mechanistic understanding of pathophysiological processes but also as an aid in the clinical decision-making process.

The use of 4D flow CMR in clinical practice is limited by long acquisition times. Scan time is usually a trade-off between spatial coverage and spatiotemporal resolution or signal-to-noise ratio. To overcome this trade-off, acceleration techniques have been developed that undersample k-space and rely on spatiotemporal redundancy in the data to complete the image reconstruction process. Parallel imaging, k-t PCA, k-t sensitivity encoding (SENSE), k-t generalized autocalibrating partially parallel acquisition (GRAPPA), and compressed sensing permit acceleration factors that have made 4D flow MRI clinically feasible [6], [7], [8], [9], [10], [11], [12], [13]. However, with these techniques image quality can be deteriorated by residual aliasing artefacts, noise, and blurriness, as well as underestimation of peak velocities and flow volumes [10], [11], [14], [15], [16].

Another way to reduce scan time is to exploit the redundancy that exists in the encodings used to obtain three-directional velocity data. 4D flow MRI is conventionally acquired with three motion encodings and one reference encoding [17]. In this 4-point scheme, the phase of the reference encoding is subtracted from the phase of the motion encodings to correct for phase errors shared by all encodings caused by effects such as inhomogeneities in the static magnetic field and eddy currents [18]. While 4D flow CMR is usually based on spoiled gradient-echo sequences, the refocusing properties of balanced steady state free precession (bSSFP) can be exploited to measure three-directional velocity data without the reference encoding [19]. Referenceless 4D flow limits the necessary motion encodings to three and thereby reduces the amount of data to acquire by 25%. However, bSSFP sequences are more susceptible to off-resonance effects, which limit their applicability for 4D flow [19]. Another approach is needed to achieve referenceless 4D Flow for spoiled gradient echo sequences, which standard 4D flow sequences are built on.

Deep learning represents a promising approach to identify patterns in MR images and may be useful in identifying the phase-offset field directly from the velocity-encoded acquisitions, thereby achieving referenceless 4D flow for spoiled gradient echo sequences. This approach has been investigated previously for 4D flow of the brain, where a deep learning model was trained to reconstruct the three-directional velocity data from the three motion encodings, showing an excellent agreement between the deep learning-based referenceless and the conventional 4-point approach [20]. The scan time reduction gained by deep learning-enabled referenceless 4D flow is not associated with increased noise and artefacts that typically affect undersampling-based acceleration techniques.

While referenceless 4D flow with deep learning has been investigated for brain magnetic resonance imaging (MRI), cardiovascular applications have specific challenges. First, the phase errors common to all encodings have a temporal evolution due to cardiac motion. Second, the lungs typically constitute a large area with noise at the center of the image and close to the cardiovascular regions of interest. Third, the heart and main blood vessels host a broad spectrum of velocities, due to high pulsatility. High velocities are important for the clinical assessment of valve stenosis, while low velocities are important for estimation of wall shear stress or left atrium stasis assessment. Finally, turbulent kinetic energy (TKE), a promising marker for several cardiovascular diseases that can be estimated from the magnitude of the 4D flow CMR signal, has not yet been investigated for referenceless 4D flow [21], [22]. Consequently, 4D flow CMR would benefit from a referenceless approach that can predict not only the three-dimensional (3D) corrected velocity field across a broad range of velocities in the presence of noise but also the magnitude of the reference encoding, allowing for the estimation of TKE.

The initial approach to referenceless deep learning-enabled 4D flow of the brain used a U-Net [23], which is a type of convolutional neural network particularly successful in image-to-image translation and segmentation tasks [20], [23], [24], [25], [26], [27]. This approach required empirical velocity-based tuning of the loss function to overcome the problem of uneven class distribution, as the relevant velocity information is contained in the vessels that consist of relatively small regions compared to the entire CMR image. Referenceless 4D flow constitutes an image-to-image translation problem that seeks to map the uncorrected motion-encoded images to the reference image. Such image-to-image translation problems are suitable for conditional generative adversarial networks (cGAN) [28]. A cGAN consists of the following two networks: a generator that learns to predict output images as close as possible to the ground truth, and a discriminator, which learns to classify the images as real (ground truth) or fake (predicted by the generator). This competitive process is referred to as adversarial learning. Compared to an original GAN, conditional information is added to the generator and the discriminator in a cGAN, which is typically an image in image-to-image translation problems. By learning features in the image that make them real, the role of the discriminator may make the cGAN suitable for referenceless 4D flow CMR with deep learning without empirical tuning of the loss function.

The aim of this study was therefore to develop referenceless 4D flow CMR with deep learning for cardiovascular applications, hereby predicting the reference encoding from the three motion encodings. In addressing this aim, we trained a U-Net model with adversarial learning and with the loss function tuned by the velocity frequency similar to Kim et al. [20], and compared to the conventional 4-point approach where the reference encoding is acquired at the scanner.

## 2. Methods

### 2.1. Subjects and data acquisition

A total of 169 4D flow datasets acquired between 2013 and 2015 were retrospectively included. The subjects had different types and grades of cardiomyopathies, including chronic ischemic heart disease, idiopathic dilated cardiomyopathy, diastolic heart failure, mild-to-moderate aortic valve stenosis and mild-to-moderate mitral valve regurgitation. The study was approved by the regional ethical review board and all subjects gave written informed consent prior to participating. All research activities were performed in accordance with the Declaration of Helsinki.

Free-breathing, respiratory navigator gated, retrospective vector cardiogram controlled cardiac gated, Cartesian 4D flow MRI with a 4-point motion encoding scheme were acquired on a 3T scanner (Philips Ingenia, Philips Healthcare, Best, the Netherlands, software release R4.1.2). A non-symmetric motion encoding scheme with three motion encodings (1,0,0), (0,1,0), (0,0,1), and one motion-compensated reference (0,0,0) was used. An anterior coil consisting of 16 channels was used for the acquisition. 4D flow was acquired immediately after injection of gadolinium contrast agent (0.2 mmol/kg Gadovist, Bayer Schering Pharma AG, Berlin, Germany), prior to a late-enhancement study. Scan parameters included a sagittal-oblique slab covering the whole heart and the thoracic aorta with a 3D field-of-view = 283 – 360 × 283 – 360 × 106 – 168 mm^3^ and matrix size = 112 – 192 × 112 – 192 × 38 – 60, velocity encoding limit (VENC) 120 (cm/sec), flip angle = 10°, echo-time = 2.6 ms, repetition time = 4.3 ms, k-space segmentation factor = 3, effective acquired temporal resolution = 53 ms, reconstructed to 40 timeframes, acquired and reconstructed spatial resolution ∼2.8 × 2.8 × 2.8 mm^3^, elliptical k-space acquisition and SENSE = 3 in anterior-posterior direction. Scan time was approximately 8–12 min with the respiratory navigator gating, which had a window of 4 mm for the inner 20% of k-space and an outer window of 15 mm.

### 2.2. Data preparation

Correction for concomitant gradient fields was performed on the scanner, and for each subject, the magnitude and the phase images of the four motion encodings were reconstructed on the scanner. The real and imaginary images were computed offline after normalization of the magnitude images between 0 and 1. The real and imaginary images of each timeframe in each subject were centralized by subtraction of the mean value. All the images were visually inspected to ensure data quality. Consequently, 14 subjects were excluded due to poor blood-tissue contrast caused by a wrong flip angle resulting in very low signal in blood, and 29 subjects that underwent sternotomy were excluded due to distortion in the static magnetic field due to a stainless-steel wire in the sternum. Of the remaining 126 subjects, 10% (N=13) was used as a test dataset and 90% (N=113) was used as training and development dataset for 5-fold cross-validation. The main characteristics of the subjects are reported in Table 1.

**Table 1:** Demographics of the subjects used for training and testing.

N Females	Age (y)	Weight (Kg)	Height (m)	BSA (m^2^)
40 (32%)	65±10	80±14	1.73±0.09	1.93±0.18

Values are reported as mean±standard deviation. *BSA* body surface area, computed with Du Bois formula

### 2.3. Model and training

**Fig. 1:**
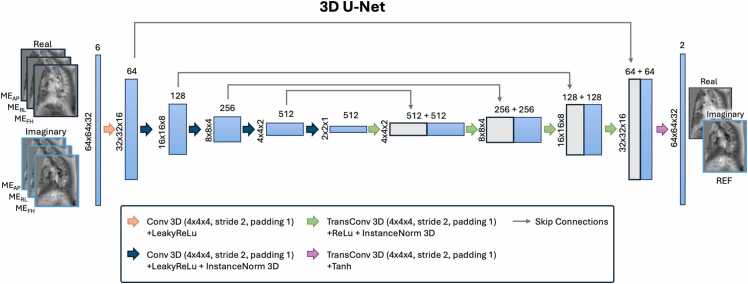
Schematic of the U-Net model used in this study. The model had a six-channel input, consisting of the real and imaginary components of the three non-corrected ME images and was trained to predict the real and imaginary components of the REF. *ME* motion-encoded, *REF* reference image

A 3D U-Net with five layers (Fig. 1) previously implemented for image-to-image translation was adapted to predict the reference encoding from the three motion encodings [28]. Rather than predicting the phase-subtracted velocity images, the model was designed to predict the complex-valued reference image, to allow estimation of both velocity and turbulent kinetic energy [21]. The model was implemented with a six-channel input, consisting of the real and imaginary images of the three motion-encoded images, and a two-channel output, i.e. the real and imaginary part for the reference image.

The 3D UNet was trained with adversarial learning (U-Net_ADV_), by integrating it as the generator in a cGAN approach, where the discriminator consisted of a convolutional network with four layers [28]. The loss function used for the generator was the sum of a mean absolute error loss and a binary cross-entropy with logits for the generated images to be classified as real. For the discriminator, a binary cross-entropy loss was used as well, and the losses for classifying fake images as fake, or real images as real were averaged.

In addition, to compare the adversarial learning to an empirical tuning of the loss function, the same 3D U-Net was trained on the same training set with an approach similar to that proposed by Kim et al. (U-Net_VEL_) [20]. The loss function was weighted with a homogeneous velocity weighting method as described by Kim et al.: the histogram of velocity magnitudes was computed over the training dataset using 99 bins from 0 to 0.3*VENC (0.4 m/s). The voxels with velocity magnitude from 0.3*VENC to 2 m/s were grouped in one bin. The weights are computed as the inverse of the velocity frequency multiplied by the magnitude image:

$$w_{i}=\frac{M}{p(v_{i})}$$

where M is the magnitude image and $p(v_{i})$ is the probability of the velocity value in the voxel i.

The training was performed using randomly selected 3D patches of size 64 × 64 × 32 voxels, from a randomly selected timeframe at each epoch, as a form of data augmentation. Additional data augmentation included random rotation, random flipping on all three axes and random zoom, and were performed on the entire volume before patch selection. The number of epochs was chosen based on when the loss function on the development dataset was not decreasing further in the first fold on the cross validation. The U-Net_ADV_ model was trained for 2500 epochs (∼ 32k iterations), with a batch size of 8. The learning rate was constant at 0.002 for 1000 epochs, and then linearly decaying to 0. The U-Net_VEL_ was trained for 8000 epochs (∼ 95k iterations). A constant learning rate of 0.002 was used for 4000 epochs, and then linearly decaying to zero. For both models, an AdamW optimizer was used. The training was performed on a workstation equipped with a 24 GB NVIDIA GeForce RTX 3090 GPU (NVIDIA, Santa Clara, California) and each training was about 2.5 h for the U-Net_ADV_, and 5 h for the U-Net_VEL_ (∼15 and ∼25 h total training time from the 5-fold cross-validation experiments, respectively). The model and training were implemented using PyTorch [29] and data augmentation was performed with MONAI [30]. The code is available at https://gitlab.liu.se/cim_public/4dflow_referenceless.

### 2.4. Post-processing and analysis of results

On the test subjects, the entire volume and all timeframes in the 4D flow data were used. The mean absolute error and the mean squared error were evaluated for the five-fold cross-validation models. Then, the predicted reference images from the deep learning models were averaged between the predictions of the five models for further evaluation. The velocity images were obtained by subtracting the phase of the reference encoding generated by the U-Net_ADV_ or the U-Net_VEL_ (“referenceless data”) and of the reference encoding reconstructed from the scanner (“conventional data”) from the phase of the motion encodings. Velocity images were automatically corrected for phase-wraps and residual background phase offset due to eddy currents with a 4th-order polynomial fitting [31], [32], [33]. Turbulent kinetic energy maps were obtained from the magnitude images as $\mathrm{TKE}=\;\frac{1}{2}\rho \sum_{i=1}^{3}\sigma_{i}^{2}$, where ρ is the blood density assumed to be 1060 kg/m^3^, and $\;\sigma_{i}^{2}=\frac{\pi }{\mathrm{VENC}}2\ln(\frac{\left|S_{i}\right|}{\left|S_{0}\right|})$ is the intra-voxel velocity variance, where ∣S_i_∣ is the magnitude image for each motion encoding direction, *i* (anterior-posterior, right-left, feet-head), and ∣S_0_∣ is the magnitude image of the reference encoding [21], [22]. Time-resolved segmentations of the 4D flow datasets in the test subjects were generated with an in-house deep learning tool or multi-atlas based method combined with manual adjustment [24], [34].

To assess the agreement between the methods for velocity mapping in the whole heart and main vessels, linear regression and Bland-Altman analysis were performed. These analyses were also performed without residual background phase offset correction, for comparison. Additionally, pulmonary flow volume (Qp) and systemic flow volume (Qs), as well as the volume-averaged (mean) and maximum velocity at peak systole, volume-integrated (total) and maximum turbulent kinetic energy (TKE) at peak systole for all cardiac chambers and major vessels were computed, and linear regression as well as Bland-Altman analysis were performed.

## 3. Results

The U-Net_ADV_ showed a lower variability between the folds, as the U-Net_VEL_ had a mean squared error of more than 10 times larger for fold 2 and 4 compared to the other folds (0.02 and 0.01 vs 0.0014 and 0.0012 and 0.005). The mean absolute error and the mean squared error between the acquired reference complex image and the complex image predicted by the U-Net_ADV_ and the U-Net_VEL_, respectively, for the five different models trained with the five-fold cross-validation are reported in Supplementary Table 1. Note that this error is computed for the entire image volume, and not solely in regions with elevated blood flow velocities for which the loss function of U-Net_VEL_ is optimized.

**Fig. 2:**
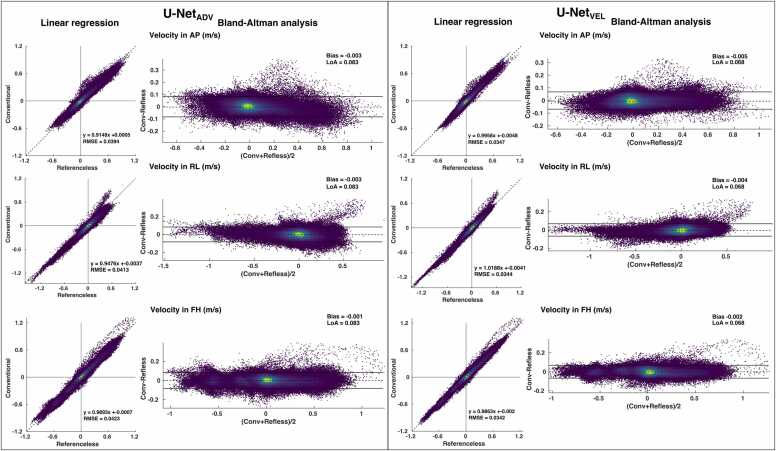
Linear regression and Bland-Altman analysis for velocities at peak systole (averaged between four timeframes) for the U-Net_ADV_ (left) and the U-Net_VEL_ (right); *LoA* limits of agreement, *AP* anterior-posterior, *RL* right-to-left, *FH* feet-to-head direction

The results of the linear regression and Bland-Altman analysis for the velocities at peak systole (averaged over four timeframes) are reported in Fig. 2 for the U-Net_ADV_ (on the left) and U-Net_VEL_ (on the right). All the voxels in the heart and main vessels for all the tests subjects are represented in the figure. In systole, U-Net_ADV_ had a bias slightly closer to 0 (−0.003 vs −0.005, −0.003 vs −0.004, −0.001 vs −0.002 m/s for anterior-posterior, right-to-left and feet-to-head directions, respectively); however, the U-Net_VEL_ showed a narrower distribution of velocity points, with slopes closer to 1 in the anterior-posterior and right-to-left direction (0.99 vs 0.91, 1.01 vs 0.95 for U-Net_VEL_ and U-Net_ADV_ respectively), narrower limits of agreements (0.068 vs 0.083 m/s for U-Net_VEL_ and U-Net_ADV_, respectively). and smaller root-mean-squared errors in the three directions.

**Fig. 3:**
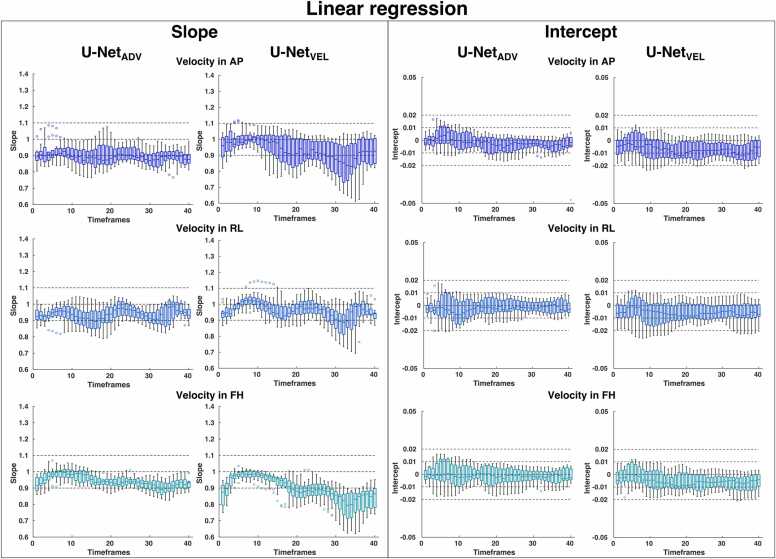
Distributions of the slope and intercept of the linear regression analysis over the test subjects reported as a boxplot at each timeframe in the three velocity directions for the U-Net_ADV_ and the U-Net_VEL_; *AP* anterior-posterior, *RL* right-to-left, *FH* feet-to-head direction

**Fig. 4:**
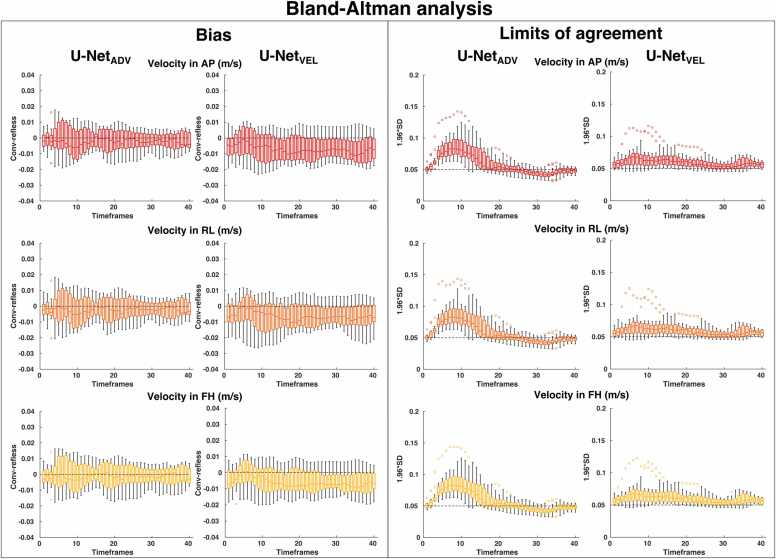
Distributions of the bias and limits of agreement of the Bland-Altman analysis over the test subjects reported as boxplot at each timeframe in the three velocity directions for the U-Net_ADV_ and the U-Net_VEL_; *SD* standard deviation, *AP* anterior-posterior, *RL* right-to-left, *FH* feet-to-head direction

The slope and intercept of the linear regression for each timeframe are reported in Fig. 3, and the bias and limits of agreement of the Bland-Altman analysis for each timeframe are reported in Fig. 4**.** The boxplots represent the distributions over the test subjects for U-Net_ADV_ (left) and U-Net_VEL_ (right) in the three velocity directions. To compress the results into single numbers, median values of the median and the interquartile range of these boxplots for the whole cardiac cycle, the systolic timeframes (1–16) and diastolic timeframes (16–40) are reported in Table 2. The U-Net_ADV_ had lower median interquartile ranges over the cardiac cycle than the U-Net_VEL_, except for the slope in the feet-head direction. U-Net_ADV_ had the slope that deviated most from identity in the anterior-posterior direction, but the highest interquartile range in the feet-head direction. On the other hand, U-Net_VEL_ had the slope that deviated most from identity in the feet-head direction and highest interquartile range in the anterior-posterior direction. The Bland-Altman bias for the U-Net_ADV_ was smaller than for the U-Net_VEL,_ and the interquartile range was similar between the models. The limits of agreement for the U-Net_ADV_ were wider in systole than in diastole, while the U-Net_VEL_ had similar limits of agreement in systole and diastole, being lower in systole, and higher in diastole compared to the U-Net_ADV_.

**Table 2:** Results of the linear regression and Bland-Altman analysis across the test subjects.

U-Net_ADV_					
		Slope	Intercept	Bias	Limits of agreement
Whole cardiac cycle	AP	0.895 / 0.07	−0.003 / 0.009	−0.002 / 0.01	0.052 / 0.009
	RL	0.939 / 0.075	−0.002 / 0.01	−0.002 / 0.011	0.052 / 0.009
	FH	0.937 / 0.048	−0.001 / 0.01	−0.001 / 0.011	0.052 / 0.009
Systole (timeframes 1–16)	AP	0.897 / 0.066	0 / 0.012	−0.002 / 0.014	0.074 / 0.022
	RL	0.927 / 0.074	−0.003 / 0.014	−0.002 / 0.015	0.074 / 0.022
	FH	0.982 / 0.052	−0.001 / 0.013	−0.002 / 0.013	0.074 / 0.021
Diastole (timeframes 16–40)	AP	0.894 / 0.072	−0.003 / 0.008	−0.002 / 0.009	0.048 / 0.008
	RL	0.95 / 0.075	−0.002 / 0.009	−0.002 / 0.01	0.049 / 0.008
	FH	0.929 / 0.047	−0.002 / 0.01	−0.001 / 0.01	0.048 / 0.008
U-Net_VEL_					
		Slope	Intercept	Bias	Limits of agreement
Whole cardiac cycle	AP	0.925 / 0.141	−0.007 / 0.013	−0.007 / 0.014	0.058 / 0.011
	RL	0.966 / 0.067	−0.006 / 0.011	−0.007 / 0.013	0.058 / 0.011
	FH	0.887 / 0.066	−0.006 / 0.011	−0.007 / 0.011	0.058 / 0.011
Systole (timeframes 1–16)	AP	0.983 / 0.084	−0.005 / 0.014	−0.005 / 0.016	0.063 / 0.014
	RL	0.982 / 0.052	−0.005 / 0.015	−0.005 / 0.015	0.062 / 0.014
	FH	0.975 / 0.035	−0.004 / 0.012	−0.004 / 0.012	0.063 / 0.015
Diastole (timeframes 16–40)	AP	0.909 / 0.161	−0.008 / 0.012	−0.008 / 0.013	0.056 / 0.01
	RL	0.956 / 0.078	−0.007 / 0.011	−0.007 / 0.012	0.056 / 0.01
	FH	0.868 / 0.075	−0.006 / 0.01	−0.007 / 0.011	0.056 / 0.01

Median values for the whole cardiac cycle, for only the systolic timeframes (1–16) and for diastolic timeframes (16–40) of the median/interquartile range across the test subject of the slope and intercept of the linear regression, as well as bias and limits of agreement of Bland-Altman analysis. *AP* antero-posterior, *RL* right-to-left, *FH* feet-head

In Supplementary Figs 1 and 2, the slopes and intercept of the linear regression, as well as the bias and limits of agreement of the Bland-Altman analysis are reported for velocities before performing correction for residual background phase offsets with 4th-order polynomial fitting. The U-Net_ADV_ had a higher bias before correction, with a median of −0.15 m/s compared to −0.002 m/s after correction over the cardiac cycle. On the other side, the U-Net_VEL_ had smaller biases before correction, with a median value of −0.002 m/s vs −0.007 m/s in systole and −0.004 m/s vs −0.007 m/s in diastole for the three velocity directions (Table 2 and Supplementary Table 2).

**Fig. 5:**
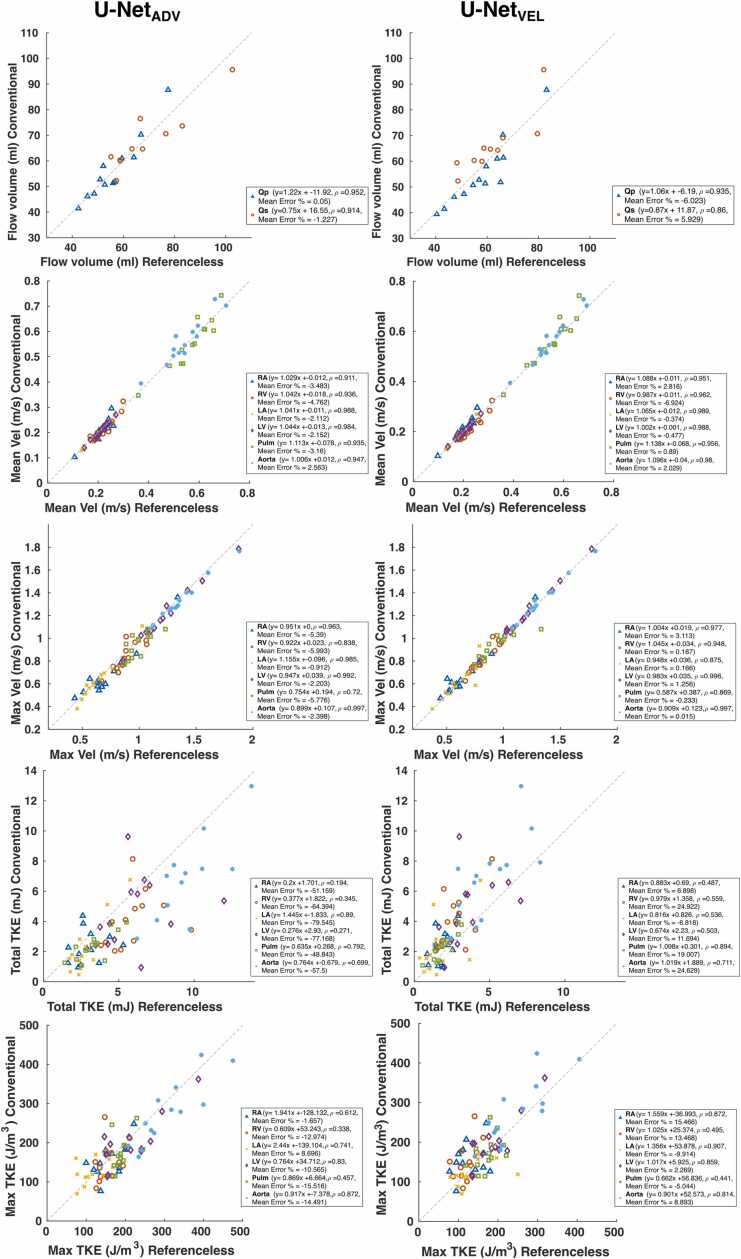
Linear regression analysis for the pulmonary and systemic flow volumes (Qp and Qs) (top row), mean (second row) and maximum velocity (third row), total (fourth row) and maximum turbulent kinetic energy (last row) at peak systole in the cardiac chambers and main vessels for the U-Net_ADV_ (on the left) and the U-Net_VEL_ (on the right). For flow volumes, three subjects were excluded from the Qs calculations as the aorta was not entirely included in the field of view. *TKE* turbulent kinetic energy, *ρ* Pearson correlation coefficient, *RA* right atrium, *RV* right ventricle, *LA* left atrium, *LV* left ventricle, *Pulm* pulmonary artery

Fig. 5 shows results from the regression analysis for the pulmonary and systemic flow volumes, mean and maximum velocities and total and maximum turbulent kinetic energy. For pulmonary and systolic flow volumes, the error was 0.05% and −1.23% for the U-Net_ADV_ and −6.02% and +5.92% for the U-Net_VEL_, respectively. For mean velocity, the worst agreement was seen in the right ventricle with an error of −4.76% and −6.92% for U-Net_ADV_ and U-Net_VEL_, respectively. For the maximum velocity, the largest mean difference was −5.99% for U-Net_ADV,_ in the right ventricle, and 3.11% for U-Net_VEL_ in the right atrium. For total turbulent kinetic energy, the largest error was −79,54% in the left atrium for the U-Net_ADV_ and +24.92% in the right ventricle for the U-Net_VEL_. For maximum turbulent kinetic energy, the largest error was −15.46% in the pulmonary artery for the U-Net_ADV_ and +15.46% in the right atrium for the U-Net_VEL_. Bland-Altman analyses for flow volumes, velocities and turbulent kinetic energy are reported in Supplementary Figure 3**.** The mean bias in the flow volumes was −0.15 mL vs 0.09 mL and the limits of agreement were 10.17 vs 12.26 mL for U-Net_ADV_ and U-Net_VEL_, respectively. For mean velocities, the bias was −0.003 vs 0.002 m/s and the limits of agreement were 0.046 vs 0.038 m/s for U-Net_ADV_ and U-Net_VEL_, respectively. For maximum velocities, the bias was −0.03 vs 0.01 m/s and the limits of agreement were 0.06 and 0.11 m/s for U-Net_ADV_ and U-Net_VEL_, respectively. For the total turbulent kinetic energy, the bias was −1.42 vs 0.98 mJ, while the limits of agreement were 3.76 vs 3.06 mJ, for U-Net_ADV_ and U-Net_VEL_, respectively. For the maximum turbulent kinetic energy, the bias was −2.14 vs 18.31 J/m^3^ and the limits of agreement 145,41 and 105.56 J/m^3^ for U-Net_ADV_ and U-Net_VEL_, respectively.

**Fig. 6:**
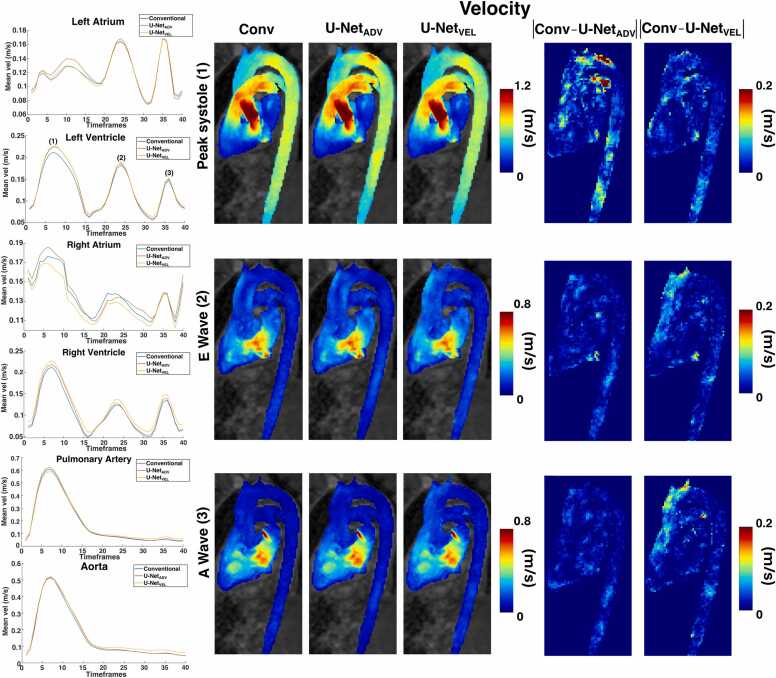
On the left: mean velocity curves in the cardiac cycle in the heart chambers and main vessels for one representative subject. On the right: velocity maximum intensity projections and absolute difference maps at three timeframes in the cardiac cycle: peak systole (1), peak of the E wave (2) and peak of the A wave (3)

**Fig. 7:**
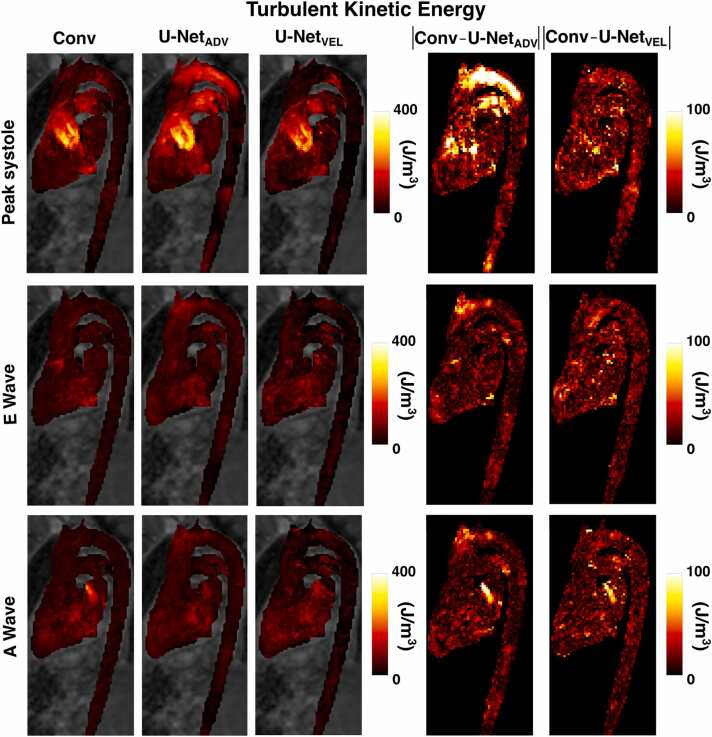
TKE maximum intensity projections and absolute difference maps at three timeframes in the cardiac cycle: peak systole, peak of the E wave and peak of the A wave

In Fig. 6, mean velocity curves in the cardiac chambers and main vessels are reported on the left, while maximum intensity projection images and absolute difference maps for velocities at three timeframes are reported on the right for one representative subject. In Fig. 7, maximum intensity projection images and absolute difference maps for TKE at three timeframes are reported for the same representative subject. The three timeframes are peak systole, peak early (A wave), and peak late (E wave) ventricular filling. For this subject, both U-Net_ADV_ and U-Net_VEL_ show overall good agreement with conventional 4D flow. U-Net_ADV_ overestimated velocities and TKE in the aortic arch at peak systole, while both models underestimated velocities in the distal descending aorta. U-Net_VEL_ overestimated the velocities in the distal ascending aorta both at the peak of the A wave and the E wave. Supplementary Fig. 4 shows maximum intensity projections and absolute difference maps of velocities and turbulent kinetic energy at peak systole for other two representative subjects in the test dataset. The outliers seen in the regression and Bland Altman-analysis Fig. 2 are voxels in the aortic arch of Subject 3 in Supplementary Fig. 4.

Supplementary Fig. 5 shows the residual phase offset for the same representative subject as in Fig. 6 and Fig. 7 in one sagittal slice in the middle of the field of view. U-Net_ADV_ has a residual phase offset that is smoother and more similar to that in conventional 4D flow, when compared to U-Net_VEL_.

## 4. Discussion

We demonstrated that three-directional velocity data for 4D flow CMR in cardiovascular applications can be obtained with a reference encoding predicted from the three motion encodings by using deep learning. Overall, there was good agreement between 3-point referenceless 4D Flow and conventional 4-point 4D flow, both for a U-Net trained with adversarial learning and the same U-Net trained with a loss function weighted by the inverse of the velocity frequency distribution. U-Net_ADV_ performed more consistently throughout the cardiac cycle and across the test subjects. On the other hand, U-Net_VEL_ performed better for systolic velocities, voxel-wise values and maximum and mean velocities across the region of interest. Moreover, the U-Net_VEL_ systolic velocity and TKE maximum intensity projections were visually more similar to those computed from conventional 4D flow data, when compared to U-Net_ADV_. Estimation of total turbulent kinetic energy was poor for both models, while results on maximum turbulent kinetic energy were promising.

Adversarial learning seems to predict a reference which has lower inter-subject variability in the test set compared to the empirical tuning of the loss function by the inverse of velocity frequency. The velocity-based weighting of the U-Net’s loss function was introduced for referenceless 4D flow in the brain to overcome the problem of uneven class distribution towards voxels with low velocities, which more frequent in the training dataset as the brain composed of small vessels surrounded by a large area with null velocities [20]. However, we speculate that the inter-subject variability of flow velocities in the heart and main vessels may be higher compared to the variability of flow velocities in organs such as the brain. The subjects used for training had different types of cardiomyopathies, which implies different flow characteristics for different subjects. Therefore, the homogenous weighting of velocities inside the lumen may be less applicable in cardiovascular applications, which could explain why U-Net_ADV_ appears to generate results that are more stable across the subjects than U-Net_VEL_, throughout the cardiac cycle. Further, U-Net_VEL_ had more variability in the fold of the cross-validation evaluation. Such variation in the training data may affect the performance of the weighted U-Net_VEL_ more than the U-Net_ADV_, which had a constant loss between the folds, thus showing a more robust behavior for the training data used. U-Net_ADV_, on the other hand, generated erroneous areas with elevated velocity and turbulent kinetic energy in systole, which are less frequently seen when weighting the loss function by the inverse of velocity frequency in U-Net_VEL_. Both models performed poorly for a subject with severe aortic stenosis and high velocities in the ascending aorta, indicating that both models fail to generalize beyond the distribution in the training set. Including more patients with a larger range of velocities in the training set could help generalizing the models.

In this study, the correction of residual background phase offsets due to eddy currents is not included in the model. Instead, residual background phase offsets were corrected in the predicted velocity images, as in standard 4D flow workflows. This was done with an automated background correction method based on a 4th-order polynomial fit to the static tissue [33]. Counterintuitively, U-Net_VEL_ showed a larger bias after background phase offset correction. We speculate that the velocity-based weighting of the loss function de-emphasizes the prediction of overall image features and makes the reference encoding predicted by U-Net_VEL_ more different than the true reference encoding in non-weighted regions of the image when compared to U-Net_ADV_. Thus, residual background phase offset correction with polynomial fitting in the static tissue may not be suitable for more artificial residual background phase offset in U-Net_VEL_. This might be solved by embedding the correction for residual offset in the network. Future studies should evaluate the best strategies to optimize residual offset correction for U-Net_VEL_.

Predicting the complex-valued reference encoding from the three motion encodings, rather than predicting the phase-subtracted velocity image, permits estimation of the intravoxel velocity standard deviation and turbulent kinetic energy from the magnitude of the complex MR signal [21], [22]. The agreement between turbulent kinetic energy obtained with deep learning-enabled referenceless and conventional data was inferior to the agreement seen for velocity. When compared to the role of the reference image in velocity mapping, where effects of inhomogeneities in the static magnetic field and certain eddy currents are corrected for by phase-subtraction, turbulence mapping is a magnitude-based application where the effects of factors other than flow that affect the signal amplitude, such as spin density and relaxation effects, are corrected for by taking the quotient in signal magnitude between the flow encodings and the reference encoding. We speculate that larger training sets may help the networks to better learn these effects on the magnitude images, and that data with more voxels affected by turbulent flow can aid the network in separating these effects from the effects related to turbulent flow. Nevertheless, the agreement for maximum turbulent kinetic energy in the aorta and left ventricle was promising and suggested that future studies should focus on optimizing the deep learning network for turbulence estimation.

Long scan time is one of the main limiting factors for the clinical use of 4D flow CMR [1]. With the deep learning-enabled referenceless 4D Flow CMR, a time-resolved reference encoding can be predicted in ∼20 s per subject. By avoiding the acquisition of the reference encoding, for a 4D flow sequence with a k-space segmentation factor of 2 and repetition time of 4 ms, referenceless 4D flow could either be used to improve the temporal resolution from 32 ms to 24 ms or, by increasing the k-space segmentation factor to 3, to reduce the scan time by 33% with a temporal resolution of 36 ms. Long scan times not only increase patient discomfort and clinical costs but also affect image quality due to patient movement during the image acquisition. Further, deep learning-enabled referenceless 4D flow CMR could potentially be combined with other acceleration techniques, such as compressed sensing.

## 5. Limitations

The main limitations of this study relate to the number of datasets and the retrospective study design. First, the number of subjects in the study is rather small, impairing the generalizability of the network as well as the evaluation of the results, as the number of subjects in the test dataset was too small to perform statistical testing. Second, all 4D flow data were retrospectively obtained from a dataset acquired with conventional 4-point encoding and subsequently decimated to the referenceless 3-point scheme. Additionally, three subjects had to be excluded from the evaluation of aortic flow because the ascending aorta was outside the field of view. Moreover, the subjects were acquired with the same encoding scheme (non-symmetric 4-point), in a single institution and on a single scanner. Future studies should include training data from different scanners, institutions, and vendors to validate the generalizability of deep learning-enabled referenceless 4D flow CMR. Finally, in this paper, we did not investigate how noise is distributed in the predicted reference encoding. Future studies should assess the effect of referenceless 4D flow CMR on the signal-to-noise ratio of the velocity and turbulence images. Future studies should also prospectively validate referenceless 4D flow CMR on 3-point data acquired without a reference encoding.

## 6. Conclusions

Referenceless 4D flow CMR enabled by deep learning agrees well with conventional 4-point 4D flow in assessing velocities and flow volumes in the heart and main vessels.

These encouraging findings suggest that deep learning-enabled referenceless 4D flow CMR could be used to reduce scan time or increase temporal resolution, aiding the use of 4D flow CMR velocity mapping in clinical practice.

## Funding

This work was supported by ALF Grants, Region Östergötland, the Medical Faculty at Linköping University, and the Analytic Imaging Diagnostics Arena (AIDA) at Linköping University.

## Author contributions

**Chiara Trenti:** Writing – original draft, Visualization, Validation, Software, Methodology, Formal analysis, Conceptualization. **Ylipaa Erik:** Writing – review & editing, Software, Methodology. **Tino Ebbers:** Writing – review & editing, Resources. **Carl-Johan Carlhäll:** Writing – review & editing, Data curation. **Jan Engvall:** Writing – review & editing, Data curation. **Petter Dyverfeldt:** Writing – original draft, Supervision, Resources, Funding acquisition, Conceptualization.

## Declaration of competing interests

The authors declare that they have no known competing financial interests or personal relationships that could have appeared to influence the work reported in this paper.

## Data Availability

The code used to train the models in this study is available at https://gitlab.liu.se/cim_public/4dflow_referenceless. The trained models are available upon reasonable request to the corresponding author.
