# Supplementary material for: Referenceless 4D flow cardiovascular magnetic resonance with deep learning
Source: J Cardiovasc Magn Reson. 2025 Jun 2;27(2):101920. doi: 10.1016/j.jocmr.2025.101920 (PMC12270065; doi:10.1016/j.jocmr.2025.101920)
Supplement: Supplementary file 1 — Supplementary material [file mmc1.docx]

**Supplementary tables**

***Supplementary Table 1*** *Mean absolute error (MAE) and mean squared error (MSE) between the acquired reference complex image and the one produced by the U-Net_ADV_ and the U-Net_VEL_.*

|  |  | **Fold 1** | **Fold 2** | **Fold 3** | **Fold 4** | **Fold 5** | **Average**± **Std dev** |
| --- | --- | --- | --- | --- | --- | --- | --- |
| **U-Net_ADV_** | MAE | 0.0107 | 0.0116 | 0.0128 | 0.0090 | 0.0108 | 0.0110±0.0012 |
|  | MSE | 0.0004 | 0.0005 | 0.0005 | 0.0003 | 0.0004 | 0.0004±0.0001 |
| **U-Net_VEL_** | MAE | 0.0277 | 0.0580 | 0.0259 | 0.0471 | 0.0419 | 0.0401±0.0121 |
|  | MSE | 0.0014 | 0.0209 | 0.0012 | 0.0100 | 0.0051 | 0.0077±0.0073 |

***Supplementary Table 2*** *Results of the linear regression and* *Bland-Altman analysis across the test subjects without correction for residual background phase offset with a 4^th^ order polynomial fitting.*

| **U-Net_ADV_** | | | | | |
| --- | --- | --- | --- | --- | --- |
|  |  | **Slope** | **Intercept** | **Bias** | **Limits of agreement** |
| **Whole**  **cardiac cycle** | AP | 0.893 / 0.069 | -0.021 / 0.008 | -0.015 / 0.008 | 0.052 / 0.01 |
|  | RL | 0.946 / 0.067 | -0.012 / 0.008 | -0.015 / 0.008 | 0.052 / 0.01 |
|  | FH | 0.939 / 0.055 | -0.014 / 0.008 | -0.015 / 0.008 | 0.052 / 0.01 |
| **Systole**  **(timeframes 1-16)** | AP | 0.9 / 0.063 | -0.018 / 0.01 | -0.015 / 0.01 | 0.075 / 0.02 |
|  | RL | 0.934 / 0.067 | -0.011 / 0.009 | -0.015 / 0.01 | 0.075 / 0.021 |
|  | FH | 0.978 / 0.052 | -0.013 / 0.009 | -0.015 / 0.01 | 0.075 / 0.02 |
| **Diastole**  **(timeframes 16-40)** | AP | 0.887 / 0.07 | -0.021 / 0.007 | -0.016 / 0.008 | 0.047 / 0.008 |
|  | RL | 0.954 / 0.067 | -0.013 / 0.008 | -0.016 / 0.008 | 0.047 / 0.008 |
|  | FH | 0.929 / 0.057 | -0.014 / 0.008 | -0.016 / 0.008 | 0.047 / 0.008 |
| **U-Net_VEL_** | | | | | |
|  |  | **Slope** | **Intercept** | **Bias** | **Limits of agreement** |
| **Whole**  **cardiac cycle** | AP | 0.952 / 0.112 | -0.007 / 0.008 | -0.004 / 0.005 | 0.056 / 0.014 |
|  | RL | 0.972 / 0.065 | -0.003 / 0.006 | -0.004 / 0.005 | 0.056 / 0.014 |
|  | FH | 0.926 / 0.074 | -0.003 / 0.005 | -0.004 / 0.005 | 0.056 / 0.014 |
| **Systole**  **(timeframes 1-16)** | AP | 0.992 / 0.07 | -0.004 / 0.006 | -0.002 / 0.006 | 0.06 / 0.015 |
|  | RL | 0.992 / 0.052 | -0.001 / 0.007 | -0.002 / 0.006 | 0.06 / 0.016 |
|  | FH | 0.994 / 0.042 | -0.001 / 0.006 | -0.002 / 0.006 | 0.06 / 0.015 |
| **Diastole**  **(timeframes 16-40)** | AP | 0.945 / 0.126 | -0.008 / 0.009 | -0.004 / 0.004 | 0.054 / 0.012 |
|  | RL | 0.97 / 0.074 | -0.003 / 0.006 | -0.004 / 0.004 | 0.054 / 0.012 |
|  | FH | 0.903 / 0.084 | -0.003 / 0.004 | -0.004 / 0.004 | 0.054 / 0.012 |

Median values in the whole cardiac cycle, in the systolic timeframes (1-16) and in the diastolic timeframes (16-40) of the median / interquartile range across the test subject of the slope and intercept of the linear fit, as well as bias and limits of agreement of Bland-Altman analysis.

**Supplementary Figures**

***Supplementary Figure 1*** *Distributions of the slope and intercept of the linear regression analysis over the test subjects reported as boxplots at each timeframe in the three velocity directions for the U-Net_ADV_ and the UNet_VEL_ without correction for residual background phase offset with a 4^th^ order polynomial fitting; AP = anterior-posterior, RL = right-to-left, FH = feet-to-head direction.*

***Supplementary Figure 2*** *Distributions of the bias and limits of agreement of the Bland-Altman analysis over the test subjects reported as boxplots at each timeframe in the three velocity directions for the U-Net_ADV_ and the UNet_VEL_ without correction for residual background phase offset with a 4^th^ order polynomial fitting; AP = anterior-posterior, RL = right-to-left, FH = feet-to-head direction.*

***Supplementary Figure 3*** *Bland-Altman analysis for flow volumes, mean and maximum velocity at peak systole, and total and maximum turbulent kinetic energy at peak systole in the test subjects.*

*
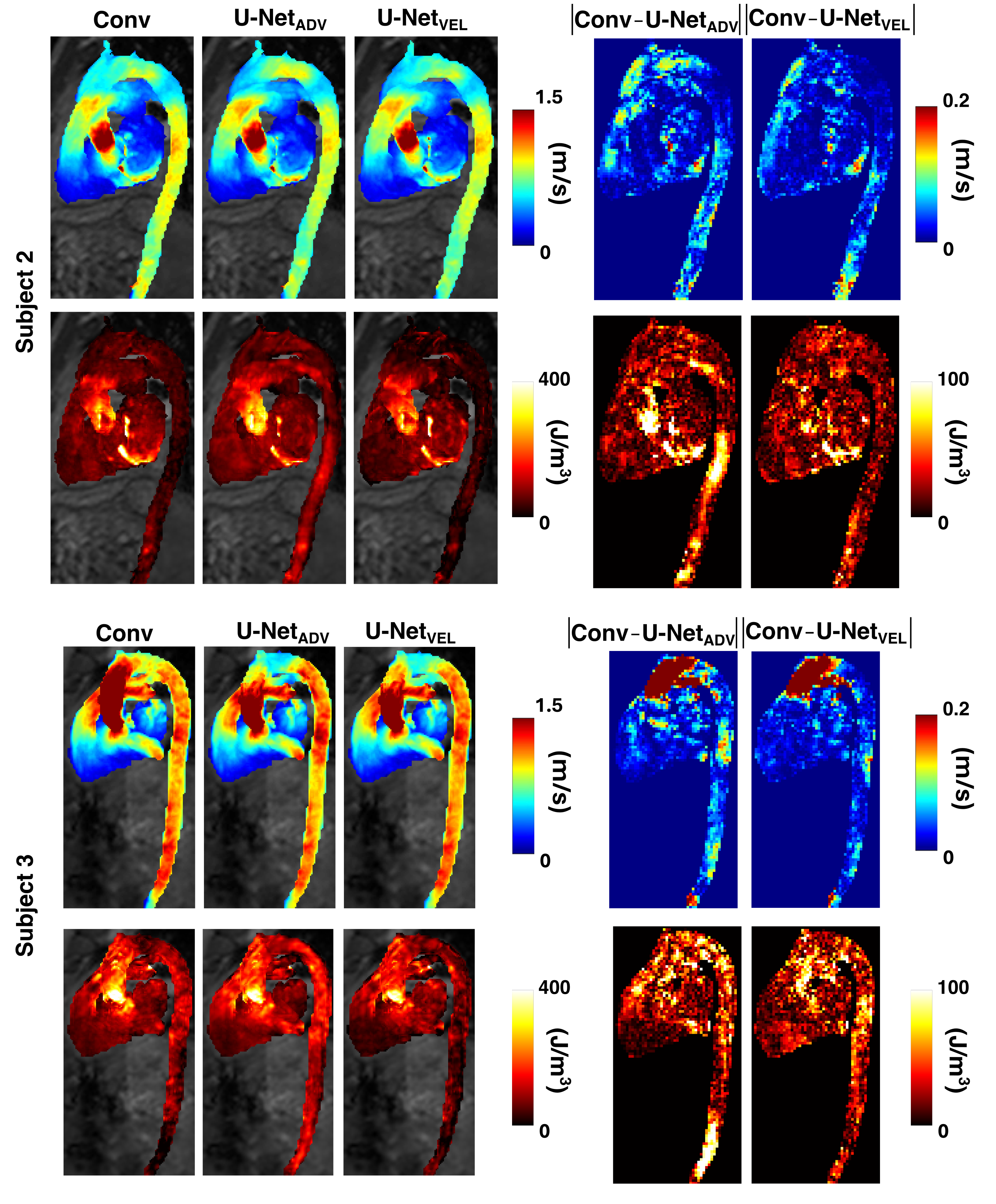
*

***Supplementary Figure 4*** *Maximum intensity projection images of velocity and turbulent kinetic energy at peak systole for two representative subjects in the test dataset.*


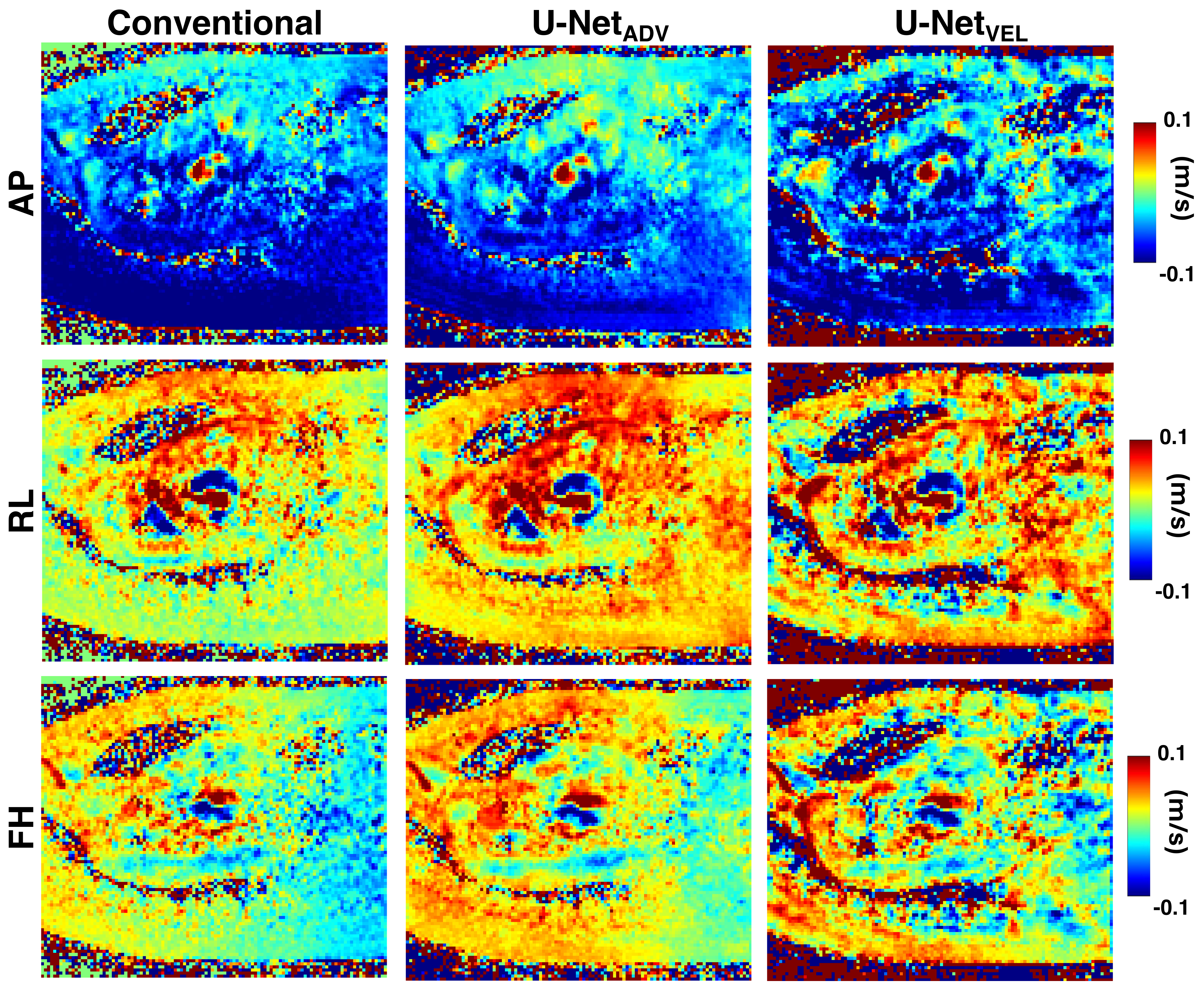


***Supplementary Figure 5*** *Residual phase offset for one representative subject in one sagittal slice in the middle of the field of view. The residual offset is smoother and more similar between the U-Net_ADV_ (centre) and the conventional 4D Flow (left) when compared to the U-Net_VEL_ (right); AP = anterior-posterior, RL = right -left, FH = feet-head direction.*
